# Systematic review—understanding the barriers and facilitators experienced by healthcare professionals in providing care for tics: a mixed methods systematic review of clinical knowledge, attitudes, and practices

**DOI:** 10.1186/s12909-024-06369-z

**Published:** 2024-11-30

**Authors:** Adam Parker, Blandine French, Madeline J. Groom, Charlotte L. Hall

**Affiliations:** 1 School of Medicine, Academic Unit of Mental Health and Clinical Neurosciences, Institute of Mental Health, University of Nottingham, Nottingham, UK; 2 NIHR MindTech MedTech Co-Operative, Institute of Mental Health, School of Medicine, Mental Health and Clinical Neurosciences University of Nottingham, Nottingham, UK; 3 NIHR Nottingham Biomedical Research Centre, Institute of Mental Health, University of Nottingham, Nottingham, UK; 4 School of Psychology, University of Nottingham, Nottingham, UK

**Keywords:** Tic disorders, Tourette syndrome, Attitudes, Beliefs, Experiences, Healthcare professionals

## Abstract

Tic disorders (TDs) have a prevalence of approximately 1%, and while various therapies have demonstrated effectiveness, people with tics and their families report a lack of understanding by healthcare professionals (HCPs). This systematic review included 13 papers featuring HCPs, people with tics, and their families about their experiences of either delivering or accessing care for tics, which were then divided into three themes. The first theme, *Need for Education and Effective Implementation*, suggested that while HCPs demonstrated good knowledge of tics, patient experiences indicated that this did not always translate to the care they received, potentially due to low confidence and a lack of formal training. The second theme, *Misinterpretation, Misdiagnosis and Stigma*, suggested that HCPs held incorrect beliefs about tics and would misinterpret tic symptoms, while caregiver responsibility for their child’s tics would both positively and negatively impact care-seeking behaviour, partly depending on their cultural belief system. Finally, the *Communication between Healthcare Professionals and Families* revealed that poor communication of a tic disorder diagnosis can exacerbate anxiety within the family. The themes of this review were demonstrated universally, demonstrating that HCPs around the world should be encouraged to understand tics and tic disorders confidently to improve the healthcare outcomes and experiences of those living with tics.

**Supplementary Information:**

The online version contains supplementary material available at 10.1186/s12909-024-06369-z.

## Highlights

• Healthcare experiences for tics have not changed substantially over the past 30 years

• Professionals lack the confidence to support patients with tics

• Complex language impedes the development of clinical relationships with families

• Tics are stigmatised in many cultures by healthcare professionals (HCPs) and their families

• Further training on tic disorders for HCPs is recommended to improve outcomes.

**Supplementary Information:**

The online version contains supplementary material available at 10.1186/s12909-024-06369-z.

## Introduction

Tics are repetitive movements, vocalisations, or sounds [1] that can be grouped into motor and phonic (or vocal) tics and include blinking, head jerking, throat clearing, humming, or repeating other people’s movements (echopraxia) and vocalisations (echolalia) [2]. There are three primary tic disorders (TDs). Provisional tic disorder refers to the experience of either motor or vocal tics for less than a year, whereas a chronic tic disorder diagnosis is given to those experiencing either tic type for longer than a year. The third, Tourette Syndrome (TS) is the most well-known and requires an individual to have experienced at least one vocal tic and two motor tics for at least one year [3, 4]. For all primary tic disorders, it is necessary for someone to have experienced tics before age 18 for them to be diagnosed, even if they first approach their GP in adulthood [4, 5]. Research suggests that in children and young people aged between 5 and 18 years, TS has an overall prevalence of approximately 1% [6] or between 4 and 8 cases in every 1000 school children [7]. Epidemiological studies of TS also suggest a higher prevalence in boys, with research indicating a male:female ratio of 4:1 [2, 8]. Attention deficit/hyperactivity disorder (ADHD) has been shown to co-occur in up to 60% of TS cases [9, 10], and between 20 and 60% of TS cases meet the diagnostic criteria for obsessive‒compulsive disorder (OCD) [9, 11].

Few people experience a complete remission of their tics between childhood and adulthood, with most reporting a decline in frequency and severity as they age [12], whereas others find that the severity and frequency of their tics persist into adulthood [8, 12]. Consequently, this has been shown to impact various quality-of-life domains, including social and familial relationships [13], employment [14, 15], stigma [16], and mental health [17], including an increased risk of suicide [18].

In the UK, support for tics typically starts with an initial assessment by a general practitioner (GP) within the primary care service of the National Health Service (NHS) [19]. The GP often makes a referral to secondary care services for specialised support from a neurologist, psychiatrist, specialist nurse or psychologist [19]. Alpha-2 agonists are usually commenced as a first line pharmacological treatment for TDs including clonidine which may assist in the management of co-occurring ADHD symptoms [20–22].

Antipsychotics such as risperidone and aripiprazole may be explored as further treatment options and while considered to be most effective for the treatment of tics, can have various adverse side effects [23, 24]. Behavioural therapies such as habit reversal therapy (HRT) [25] feature a combination of relaxation training, tic monitoring and awareness training to support the suppression of tics, with a reported reduction in tic severity ranging from 18–38% [26, 27]. Exposure and response prevention (ERP) centres around intercepting and breaking the association between the premonitory urge and post-tic relief [28]. Comprehensive Behavioural Intervention for Tics (CBIT) incorporates HRT with relaxation techniques and psychoeducation while promoting social support [28–31], and has demonstrated significant effectiveness in reducing tic severity [27–29].

Despite the various treatment options available for tic disorders, access to services is challenging [32]. In the UK, the reasons for this include a lack of specialist tic-trained therapists [32, 33] and a lack of understanding of tics, TDs, and the referral pathway among health care professionals (HCPs) [19, 34].

To improve access to healthcare services for people with tic disorders, it is important to evaluate the experiences of this population when trying to access healthcare for tics, and to understand the knowledge and understanding of tics among healthcare practitioners considering any barriers or facilitators they have experienced when delivering healthcare for those with tics. Compared with other neurodevelopmental disorders, there has been comparatively less research on the experiences of patients with tics and the perceptions of HCPs.

Currently, no other systematic review has explored the barriers and facilitators to delivering healthcare for tics and TDs experienced by HCPs, with reference to their knowledge, attitudes, beliefs, and experiences.

## Hypotheses and aims

This review aims to explore the experiences of people living with a TD when accessing healthcare services for their tics, and the barriers and facilitators of HCPs delivering healthcare for those living with tics in relation to their attitudes, beliefs, experiences, and knowledge of tic disorders.

## Materials and methods

This review was undertaken following the Preferred Reporting Items for Systematic Reviews and Meta-analysis Protocols (PRISMA-P) guidelines, and a protocol was registered with the International Prospective Register of Systematic Reviews (PROSPERO; CRD42023473483) on 18th October 2023.

### Inclusion criteria

#### Studies

Peer-reviewed and published papers of all methodologies (qualitative, quantitative, and mixed methods) were considered for this review, where the focus was on the experiences, attitudes, beliefs and perceptions of patients and HCPs in the delivery of healthcare for tic disorders. While literature reviews were not included, their reference lists were searched for relevant papers to be included.

#### Population

To comprehensively explore the published literature on the experiences of people with tic disorders and HCPs, accounts of patients with tics (e.g., children aged 2–18 years old and adults), their family members (e.g., parents, carers, and extended family) and friends, and HCPs who have directly worked with people with tics were eligible for inclusion.

#### Context

All stages in the healthcare pathway were included, e.g., primary, secondary, and tertiary, and papers were not limited by date of publication, with all studies from inception to the search date being considered.

### Exclusion criteria

Literature reviews were not included, but their reference lists were hand searched for additional papers. Case studies, theses/dissertations, opinion articles, grey literature and any papers that did not specifically discuss the experiences of patients with tics or the experiences of health professionals working with people with tics were also excluded.

### Search strategy

The MEDLINE (Ovid), PsycINFO (Ovid), and EMBASE (Ovid) databases were searched for appropriate studies. The initial search was conducted in October 2023 and was updated in July 2024. PROPSERO was also searched to identify any pre-existing systematic reviews. After the initial multipurpose search was conducted with MeSH (Medical Subject Heading) terms and subject-related words, duplicates were identified and removed (see Appendix A). Keywords related to the topic of tics (e.g., ‘tic disorder’ and ‘Tourette syndrome’), lived experience (e.g., ‘experience’, ‘perception’, and ‘knowledge’), and healthcare (‘healthcare’, ‘doctor’, ‘health professional’, and ‘patient’) were used to develop the search strategy. Manual searches of the papers included in the review were performed to identify additional texts that were suitable. Forward citation searches of relevant papers were also conducted to further discover studies that may have been missed by the search strategy.

### Study selection

**Fig. 1:**
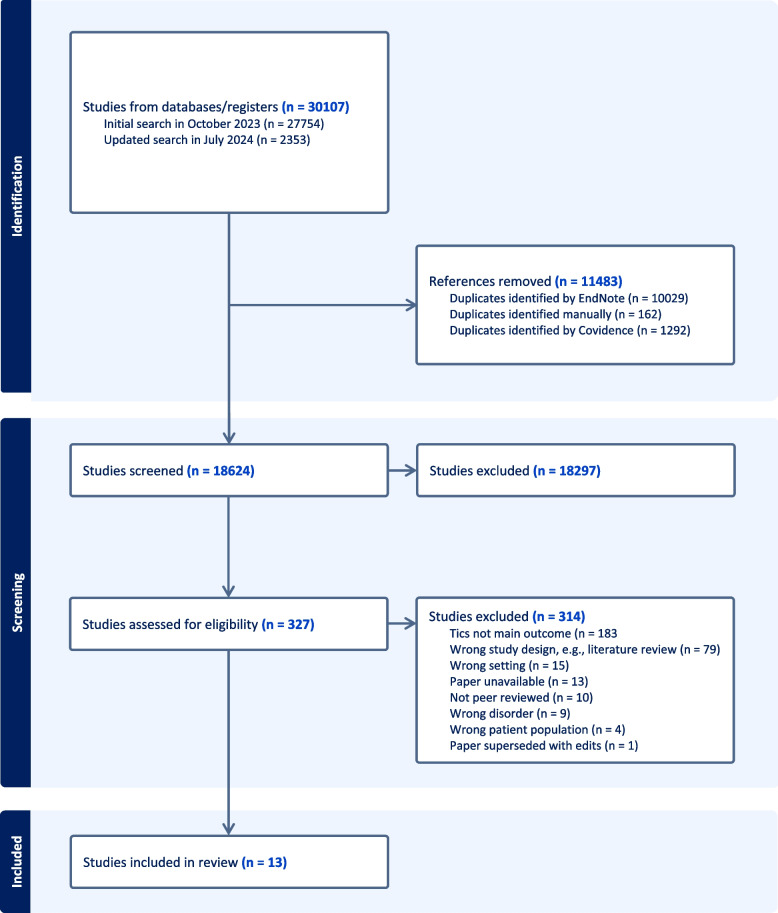
A PRISMA diagram displaying study selection process

Across the initial and updated database search, 30107 results were identified, which were imported into EndNote and deduplicated. The remaining results were then imported into Covidence for further deduplication and screening, with a breakdown shown in Fig. 1. Author AP screened titles and abstracts for all papers against the inclusion and exclusion criteria. No new papers were eligible for inclusion following the updated search in July 2024. F﻿or the full-text review, author AP screened all remaining papers with two other authors (BF and CLH) then completed an additional screening of 10% of the papers to ascertain agreement.

### Data extraction

Once the included papers were identified, a data extraction table was developed, detailing all relevant quantitative and qualitative information pertinent to the review’s aims. Using the extracted information, a coding system was developed to identify themes and subthemes across papers.

### Assessment of methodological quality

The methodological quality of the papers was assessed via the criteria by Kmet et al. [35] for qualitative and quantitative studies. For studies that used mixed methodologies, both assessments were completed. Quality scores ranging from 0 to 1 were calculated for each study. The quality scores were then classified as low (0–0.44), moderate (0.45–0.69), or high (0.70–1). A portion of the studies were assessed by a second reviewer (BF), and any discrepancies were resolved through team discussion. Seven papers scored a high rating, five scored a moderate rating, and one scored a low rating. Each paper’s classification can be seen in the study summary table.

### Data synthesis

The data were synthesised via a narrative synthesis approach to explore the experiences of HCPs to understand their attitudes, beliefs, and knowledge of tic disorders.

## Results

Appendix B summarises the results of each of the 13 papers included in this review, including the themes to which they contributed. The included studies predominantly discussed the lack of support provided by healthcare professionals, which was disseminated into three themes with further subthemes. A summary of the themes can be found in Appendix C.

### Descriptive characteristics of the included papers

The study selection process, including the reasons for exclusion, can be seen in the PRISMA diagram (Fig. 1). The included studies utilised qualitative (*n* = 2), quantitative (*n* = 7), or mixed methodologies (*n* = 4). All the quantitative and mixed studies used surveys as their methods [19, 33, 34, 36–43]. The qualitative studies used interviews or focus groups [3, 44]. A summary of the included studies can be found in Appendix B.

**Fig. 2:**
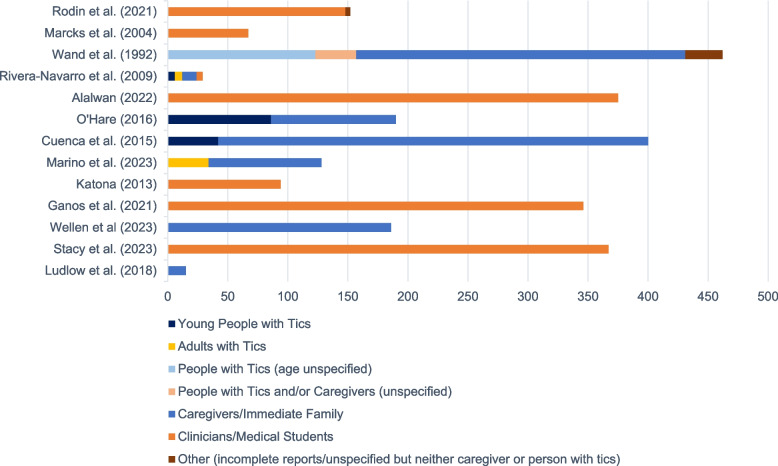
A breakdown of the types of participant samples included in each study

In this review, 73 countries were represented across all studies, with Fig. 2 displaying a breakdown of participant samples. Caregivers made up the largest participant pool (33%), followed by HCPs (32%), medical students (17%), and people with tics (10%). Males made up the majority of HCPs (60%−80%) and people with tics (65%−85%), whereas caregivers (52%−93%) and medical students (69%) were predominantly female. More information on participant demographics can be found in Appendix B, and a distribution graph of participant sample size across publication dates is included in Appendix D.

The publication dates of the included papers ranged from 1992 through 2023, with nine of the 13 papers published in the last 10 years [19, 33, 34, 36, 37, 40, 42–44] (see Appendix B and D).

The specific aims of the included studies varied, but all sought to understand the barriers to and facilitators of healthcare for tics through the experiences, attitudes, and beliefs of people living with tics, their families, or HCPs supporting people with tics.

### Summary of results

#### Need for education and effective implementation

The need for greater professional education in tic disorders was identified in all the papers and was present in all the countries. HCPs interviewed by Rodin et al. [42] commented on the inadequate quality and quantity of training provided on tics.

#### The impact of limited knowledge

Knowledge of tics was explored through symptomology, causes, diagnosis, treatments, prognosis, and comorbidities [36–39, 43]. Most HCPs scored between 60 and 80% on their understanding of tics, compared with 88% for depression, 75% for ADHD, and 57% for OCD [36, 39].

Few studies generated comparisons between care sector and occupation but of those that did HCP knowledge of tics was not significantly different from medical students’ knowledge [36], but knowledge between clinical occupations varied. Marcks et al. [39] reported no significant differences in knowledge between family physicians (primary care) and psychologists (secondary care), despite psychologists reporting seeing fewer tic cases. However, Alalwan et al. [36] reported that family physicians (primary care) and postdoctoral board-certified HCPs scored better than general practice physicians and paediatricians. Between 10 and 46% of HCPs in these studies reported working with someone with tics [36, 39], while 56% of Movement Disorder Society (MDS) members recalled seeing up to ten people with tics annually [37]. Most MDS members also reported that they did not confidently understanding tics; a finding supported by other studies in which a vast majority knew the basic definition of tics, yet only between 50 and 65% of HCPs knew the diagnostic criteria, with as few as 27% feeling confident in their understanding [36, 37, 39, 42]. The limited training opportunities available to HCPs for tics could explain the disparity between good knowledge scores and low confidence in their understanding of tics.

In studies where young people and caregivers believed that their HCP demonstrated sufficient tic knowledge, they reported feeling more confident understanding their own/their child’s diagnosis [33, 43]. Inadequate education from inexperienced lecturers left secondary care HCPs in Uganda keen to learn more about tics, particularly the identification of tics, symptoms, between-disorder differences, and treatments [42]. Other studies reported that primary care HCPs in Saudi Arabia and primary and secondary care HCPs in the USA desired to learn more about HRT as a therapy for tics [36, 39].

Secondary care HCPs often placed a greater focus on treating existing comorbidities, e.g., ADHD and autism [42, 44], and patients with comorbidities were more likely to have received medication or therapy for tics [34]. MDS members greatly endorsed treating comorbidities as an effective treatment for tics [37], and both neurologists and psychiatrists were more likely to manage co-existing diseases than to offer advice on living with tics, with 84% feeling ‘very or extremely’ confident in managing comorbidities [43].

#### Limited support and unclear referral process

Stacy et al. [43] reported that up to 86% of neurologists offered advice on living with tics, yet caregivers reported receiving limited to no information from their GP regarding support groups, managing tics, or the prognosis of tics [19, 33, 34, 39, 44], with the media or friends and family being cited as more common sources for information [41].

When presenting to primary care, people with tics and caregivers felt there was little support available [3, 19, 33, 34, 40, 44], leaving families dissatisfied with the care provided and seeing a greater number of HCPs and accessing more services overall [3, 19, 33, 34]. One study reported that, on average, families saw three HCPs for tics, with 13% reportedly seeing six or more HCPs [34]. Unfortunately, due to being unable to get support for their child’s tics, caregiver responsibilities impacted work commitments and consequently led to financial ramifications [34, 44].

Primary care HCPs demonstrated a limited understanding of available treatment options [33], often prescribing medication due to seemingly few alternatives [33, 34, 44], or having to see numerous HCPs before an alternative intervention was offered [33]. However, 69% of MDS members considered behavioural interventions to be the preferred first-line intervention for tics [37], suggesting that a lack of knowledge of these interventions may be a significant barrier for HCPs when they offer support for tics. However, Stacy et al. [43] reported that between 63 and 65% of secondary care HCPs felt confident knowing when and what treatment to commence for tics and how to manage any side effects. This difference may be explained by inconsistent approaches to tic support both nationally and internationally. Wellen et al. [34] suggested that HCPs in America were generally providing recommendations aligning with American Academy of Neurology (AAN) guidelines despite prescribing medication over behavioural therapy as first-line treatment, which is not consistent with these guidelines.

Despite a preference for behavioural therapies by people with tics and their families [33, 34, 43], an understanding of HRT and how to implement it was known only to HCPs in secondary care who were more experienced in treating tics [36, 39]; however, there was a general interest in learning more about HRT and willingness to engage with further education for tics by HCPs and medical students.

In most cases, secondary care referrals were necessary because of the limited resources available to support people with tics in primary care [19, 33, 37]. Primary care clinicians demonstrated a limited understanding of referral sources, and even those that did have knowledge, reported having little access to these services or had the referral rejected [19, 33, 37].

#### Misinterpretation, misdiagnosis and stigma

Misinterpretation of symptoms, misdiagnosis, and stigma was evident across all studies and countries in this review.

#### Misinterpretation and misdiagnosis

Primary and secondary care HCPs rated tics as ‘rare’ or ‘low’ in prevalence; however, when patients with tic presentations attended initial GP appointments, the possibility of tics were only discussed 14% of the time [3, 19, 36]. It was indicated that people presenting with tics had their symptoms misinterpreted by primary and secondary care HCP as characteristic of another disorder or comorbidity [3, 36, 42]. This suggests that the prevalence of tics may be substantially underestimated and/or overlooked, potentially due to a lack of awareness and/or training for HCPs.

In the UK, GPs reported being unsure of a tic diagnosis in approximately 6% of child children and 24% of adult cases [19], with some patients and secondary care HCPs reporting misdiagnosis of tics as another movement disorder, hyperactivity, mental illness, learning disability, brain damage, or attention-seeking behaviour [41, 42]. Nevertheless, Wellen et al. [34] reported that while 63% of parents in the UK found it difficult to find a HCP who understood tics, only 10% reported their child being misdiagnosed.

Vocal tics were more likely to be misinterpreted than motor tics [19, 41], likely because they are less common [40], and HCPs demonstrating that they are more able to recognise motor tics [42]. A large overlap in symptoms with functional tic disorder, basal ganglia disorder, and psychosomatic disorders increases the difficulty of differentiating the diagnoses [37, 42].

Marcks et al. [39] indicated that 46.1% of primary and secondary care HCPs believed that tic severity would increase in adulthood, whereas Marino et al. [19] antithetically found HCPs reported to families that symptoms would completely remit given enough time. In one study, most MDS members thought that the premonitory urge was a prerequisite of tics, whereas 28% thought that they were exclusive to chronic tics lasting longer than a year [37]. One study reported that 40% of primary care HCPs believed that coprolalia was a symptom in a majority of cases [36].

#### Stigma and misconceptions

Patients and families felt that HCPs trivialised tic symptoms [19, 33, 44], would suggest symptom exaggeration, or accuse caregivers of being overly involved with their child’s behaviour [3]. However, HCPs also suggested that some parents perceived tics as normal or stubborn traits, ultimately delaying help-seeking behaviour and furthering the impact on the child [42].

A large majority of primary and secondary care HCPs acknowledge that people living with tics experience stigma [39], with most being very or extremely concerned about this [43]. Some secondary care HCPs were also concerned about the stigma associated with the use of antipsychotics as a treatment for TDs [43], while families reported stigmatisation stemming from TD labelling and a misunderstanding of tic-related behaviour [3]. Some medical students stated that they would not want their own child to play with or, in adulthood, marry someone with tics, and others believed that people with tics should not have normal jobs like other people do [38].

Tic-related stigma was reported to be ‘pervasive’ in Ugandan families [42]. HCPs reported that cultural beliefs around witchcraft resulted in parents seeking spiritual healers in favour of healthcare support [42]. However, the same HCPs also acknowledged that parental embarrassment may eventually encourage them to seek healthcare support. In the UK, parents felt responsible for their child’s behaviour and, therefore, the reactions of other people to their child’s tics [44], resulting in families typically presenting to primary care services earlier [19]. In Spain, it was suggested that some parents were in denial when they presented with a TD diagnosis for their child, as the disorder was understood to be inherited [3].

#### Communication between healthcare professionals and families

Four of the 13 studies explored communication between healthcare professionals and families, with poor communication of a tic disorder diagnosis and complex clinical language causing anxiety within families, as they were left with more questions than answers [3].

Only 45% of secondary care HCPs valued the input of parents when making diagnostic and treatment decisions [43]. Moreover, 76% of parents valued collective decision making, with none wanting to leave all decisions solely to the HCP [43]. However, families described that the overuse of clinical language made it more difficult for families to interpret a diagnosis of TD, obstructing the ability to develop clinical relationships [3].

In the case of referrals, when families researched for available secondary care services and offered this information to GPs, it was generally well received and aided the referral process [19]. Furthermore, despite the limited support being offered, families endorsed GPs who demonstrated compassion and kept in regular contact with families regarding updates to secondary care referrals [19]. However, while it is important that parents are better informed about tics, it is noted that this may increase the demand for primary care at first onset which may not, at present, be matched by available support [34].

## Discussion

The healthcare experiences of people living with tics and the experiences of healthcare professionals in supporting people with tics have not been previously reviewed. We conducted a systematic review to explore this topic, including 13 studies covering 73 countries. The literature revealed themes that centred around the need for greater education on tics, misconceptions, misinterpretations, stigmas, and communication between HCPs and families about tics. Although these factors were predominantly barriers to accessing or offering healthcare support, a few facilitators were discussed, and the review revealed multiple areas of development to encourage better identification and management of tics.

The *Need for Education and Effective Implementation* was the most widely supported theme in the included literature, which revealed that HCPs demonstrated a level of tic knowledge akin to ADHD and better than OCD. Importantly, however, only two of the five papers [36, 39] in this review provided scores on tic knowledge among HCPs, and caution should be taken to not overgeneralise these results.

Although other common neurodevelopmental disorders, including ASD, are not discussed with the included studies, existing literature of knowledge and perceptions of ASD suggested that doctors, occupational therapists, physical therapists, and speech and language therapists demonstrated moderate to good knowledge (scores between 47–71%) [45, 46], suggesting knowledge may be similar to rates indicated in this review for tics.

Some studies found that vocal tics were more likely to be misinterpreted than motors tics [19, 41], perhaps due the perception of them being less common [40] and less recognisable [42]. Existing research supports the under recognition of vocal tics compared to motor tics [8] which can contribute to a delayed diagnosis of TS since both motor and vocal tics are necessary [8, 47]. This may therefore also translate to the diagnosis of chronic TD or provisional TD for people experiencing only vocal tics. No research included in this review compared rates of identification of simple tics versus complex tics, however, this would benefit from further research especially as some evidence does exist suggest there to be presentational differences between sex and ages [2].

Comparing the knowledge demonstrated between medical students and primary care HCPs [36], and between primary and secondary care HCPs [39], it could be suggested that knowledge of tics remains mostly unchanged between these sectors. Only the most specialised or qualified HCPs and occasionally family physicians demonstrated superior knowledge of tics. However, it is important to reinforce that not all studies sufficiently compared knowledge between groups, and further research between care sectors and clinical specialists is needed to further substantiate this trend.

Instead, limited training may hinder confidence, shaping the healthcare experiences of HCPs and people with tics internationally. French et al. [48] concluded that negative healthcare experiences of people with ADHD were indicative of a need for further education for HCPs, and the same could be suggested in this instance. Furthermore, this review revealed wide variation in national and international clinical practice for tics, which may lead some HCPs to feel better equipped to support people with tics than others. Poor confidence in tic understanding may stem from limited training opportunities to further strengthen knowledge and few opportunities for clinical supervision in this field.

Owing to limited confidence in knowledge of tics and TDs and high rates of comorbidity with ADHD and OCD [9–11], it is therefore unsurprising that HCPs more often focussed on treating comorbidities and deemed this an effective intervention for tics. While some HCPs were unfamiliar with behavioural therapies, others acknowledged their effectiveness as a tic intervention. Considering the evidence endorsing HRT [26] and CBIT [28, 30, 31, 49], it is encouraging that in some of the included studies, HCPs were prepared to learn more about available behavioural therapies [36, 39] and should be supported to further their understanding in this area to offer more targeted tic support.

Tics have been shown to impact various quality-of-life domains [13–15]. The studies included in this review highlight that difficulties accessing adequate healthcare for tics impacted family life and finances, potentially further exacerbating the impact of tics on quality of life. Whether as a result of limited knowledge of referral processes or specialists, families having to push for a referral or faced with their referral being rejected contributed to negative healthcare experiences and exacerbated the impact on quality of life. HCPs in primary care claimed to have limited access to services which may reflect a lack of tic-trained specialists, as reported by Bhikram et al. [32], indicating a need for greater provision of tic support in secondary care. There is also a need for additional training of primary care HCPs to ensure that they can identify tics and refer to secondary services appropriately.

In 1992, Wand [41] reported that 73% of participants were misdiagnosed with other movement disorders prior to receiving a TS diagnosis, with only 33% being offered any signposting support by their HCP. Comparing this data to more recent studies in 2022 and 2023, while misdiagnosis was more uncommon [34], advice on tics was still infrequent and shortcomings on HCP communication and support resulted in mostly negative experiences that have not improve substantially in over 30 years [19, 43]. Despite one paper indicating that HCPs were following the current guidelines set out by the American Academy for Neurology (AAN), this review presented findings indicating that HCPs may be underequipped to deliver assessment and treatment and that families often have negative experiences. This suggests that further guidance and HCP education is necessary to improve outcomes.

Limited training may have consequently led to misconceptions, misinterpretations, and stigma evident in 11 of the 13 papers, which were perpetuated by both HCPs and families in most countries included in this review. With respect to misidentification and misconceptions, symptomatic crossover with comorbid disorders made diagnosis difficult and impacted the confidence of HCPs. HCPs held beliefs that demonstrated inconsistencies in the understanding of tics and TDs. With respect to tic remission, while some HCPs thought that tics would completely remit and others thought that they would become more severe in adulthood, research has indicated that few people experience a complete remission; however, in most cases, tic severity declines with age [8, 12], indicating that HCPs may require additional support to reduce conflating beliefs of tic symptomology and prognosis.

Katona [38] claimed that ‘ignorance breeds fear’ when referring to medical students who held negative views of people with tics. Families alluded to the embarrassment and responsibility they felt, which had both positive and negative impacts on help-seeking behaviour, depending on culture-specific beliefs. This suggests that stigmatisation and misunderstanding of people with tics results from a lack of research and information being disseminated to HCPs and the public. Pring et al. [16] revealed that people with tics experience stigma at the individual, interpersonal, community, and structural (services and healthcare) levels, which aligns with the results of this review. It is hoped that the availability of further educational resources for HCPs may promote greater representation of people with tics and reduce stigma.

Finally, an examination of the communication between HCPs and families suggested that a shared responsibility when making care decisions was preferred by some HCPs and most families. Families preferred to make the final treatment decision, and it was important that information be conveyed without complex clinical language to avoid confusion and promote collaboration. While families perceived a lack of knowledge, they appreciated HCPs who actively followed up referral progress and displayed compassion during appointments. Therefore, HCP attentiveness was integral to the overall experience of families seeking support for tics. Improved communication of tics by HCPs to families will encourage the dissemination of knowledge and awareness, and support patients and the public to better understand tic disorders.

### Strengths and limitations

Quantitative studies were the primary methodology. Further research is needed using mixed and qualitative methods, as these methods may provide further contextual understanding of experiences than is achievable from quantitative methods alone.

This review featured studies from countries on all continents, where cultural differences may affect the perception and understanding of tics, yet professionals and families alluded to similar ideas when considering the quality of care available. However, it is important to recognise that most papers included were from Western countries, and while some perceptions might be observed internationally, further research in non-western countries is needed to ensure that the findings can also be applied to those countries. Unfortunately, it was not possible to translate non-English articles, which may have led to important data being missed. In previous research conducted by Leckman et al. [50], it was concluded that the point prevalence of tics was similar across different races. Therefore, considering the cultural belief system around witchcraft in healthcare-seeking behaviour discussed by Rodin et al. [42] in Uganda and compared with that in the West, we should be careful not to assume that additional cultural perceptions of tics would not be observed in other non-westernised cultures, further influencing help-seeking behaviours. Considering the difference between healthcare pathways internationally, it could be expected that experiences may divert significantly. However, the results indicated that the pathways of all the countries featured in this review needed improvement. Again, additional non-westernised studies may provide further clarity on pathway experiences, but it is promising that the implementation of knowledge resources may be beneficial in supporting people with tics internationally.

Most studies included in this review have been conducted since 2015 (9/13), which helps ensure that the experiences, knowledge, and attitudes discussed are more current in their representation. The reason that more recent papers exist on this subject may be related to the growing need for improvements in tic-related care. It is hoped that this review can consolidate research in this area to promote continued research necessary to better understand and improve access to care.

HCPs, people with tics, and families were included in this review. In some cases, the samples were self-selected, and owing to apparent frustration with services, they may have been more eager to engage with their respective study. As a result, participants could have experienced biases which may have impacted the accounts they provided. Moreover, HCPs who felt that they had a better understanding of tics or were more likely to see people with tics, may have been more inclined to participate.

### Implications for practice

This review demonstrated that with better implementation of knowledge resources, either pre- or post-qualification, HCPs may feel better equipped to support patients presenting with tics and dilute associated stigmas. In return, this may help families feel better understood and supported, leading to improved experiences and outcomes when accessing healthcare services.

### Implications for research

Even though some HCPs displayed good practices that were appreciated by people with tics and their families, their experiences with healthcare were generally negative and needed improvement. Future research should continue to explore international perspectives of tic disorders from healthcare professionals, people with tics, their families, and the public. This will help understand nuances that may benefit the development of resources individualised to the needs of each culture.

## Conclusion 

To conclude, this review explored the experiences of healthcare professionals in supporting people with tics and the experiences of people with tics accessing support. The findings revealed that despite a few positive service user experiences and some HCPs demonstrating good knowledge of tics, poor confidence resulting from limited training opportunities may be a barrier for other families who reported a lack of knowledgeable HCPs. This was evident across countries and had not changed substantially for over 30 years. Consequently, most families felt that HCPs were underprepared to adequately care for their patients with tics. This may have given rise to stigma and misconceptions of tics and negative experiences regarding the communication of tics and the interpretation of symptoms. Future research should continue to explore the international perspectives of tics by healthcare professionals to identify knowledge gaps and aid in resource development to better equip HCPs both new and more experienced.

## Supplementary Information

Supplementary Material 1.

## Data Availability

The data generated during the current study are available from the corresponding author upon request.

## Abbreviations

ADHD: Attention Deficit Hyperactivity Disorder
AAN: American Academy of Neurology
CBIT: Comprehensive Behavioural Intervention for Tics
ERP: Exposure and Response Prevention
GP: General Practitioner
HCP: Healthcare Professional
HRT: Habit Reversal Therapy
MDS: Movement Disorder Society
NHS: National Health Service (UK)
OCD: Obsessive–Compulsive Disorder
TD: Tic Disorder
TS: Tourette Syndrome
