# Supplementary material for: Systematic review—understanding the barriers and facilitators experienced by healthcare professionals in providing care for tics: a mixed methods systematic review of clinical knowledge, attitudes, and practices
Source: BMC Med Educ. 2024 Nov 30;24:1403. doi: 10.1186/s12909-024-06369-z (PMC11607939; doi:10.1186/s12909-024-06369-z)
Supplement: Supplementary file 1 — Supplementary Material 1. [file 12909_2024_6369_MOESM1_ESM.docx]

## Appendices

### Appendix A

*Search strategy on Ovid database.*

| 1. | exp Tics/ |  |  |  |  |
| --- | --- | --- | --- | --- | --- |
| 2. | exp Tic Disorders/ |  |  |  |  |
| 3. | ("Tourette* syndrome" or "tic disorder*" or tic or tics).mp. |  |  |  |  |
| 4. | 1 or 2 or 3 |  |  |  |  |
| 5. | (healthcare or "health care" or doctor* or "general practitioner*" or consultant* or "health professional*" or physician* or nurse* or patient*).mp. |  |  |  |  |
| 6. | (experienc* or understand* or knowledge or attitude* or perce* or view* or feeling* or belief* or thought* or phenomenology).mp. |  |  |  |  |
| 7. | (5 or 6) and 4 |  |  |  |  |

### Appendix B

*A summary of all studies included in the review.*

| Reference | Measure | Population and sample size | Quality Rating | Summary of Findings | Themes |
| --- | --- | --- | --- | --- | --- |
| Qualitative Studies | | | | | |
| Ludlow, Brown, and Schulz (2018)  UK | Semi-structured interview | 15 Parents and Caregivers   - 11 females, 4 males | High | - Parents experience many difficulties regarding tics including behaviour, reactions from other people, and financial concerns. - Parents experience misconceptions about tics by professionals, schooling staff, and the public. - Parents perceive tics to have had a negative impact on their child’s education due to lack of support or reasonable adjustments being correctly implemented. - Limited support for tics from HCPs including feeling dismissed and having little knowledge on treatment options. | *Need for Education*  *Misinterpretation, Misdiagnosis, and Stigma* |
| Rivera-Navarro, Cubo and Almanzan (2009)  Spain | Focus groups | 6 young people with tics (aged 11yrs+)   - 4 males, 2 females   6 adults with tics   - 4 males, 2 females   6 caregivers of teen with tics   - 5 females, 1 male   6 caregivers of adults with tics   - 5 females, 1 male   5 HCPs (speciality unknown)   - 4 males, 1 female | Moderate | - Diagnosis difficulty due to misinterpretation of symptoms, lack of knowledge. Lack of respect from HCPs and schooling staff. - HCPs consider the impact to depend on how much importance is placed on tics. Parents report the impact is related to peers’ lack of comprehension, rejection, and teasing. Relatives report the impact to stem from clinical language used due to lack comprehension of tics and stigmatisation. - Communication difficult due to denial within families, and the introduction of social services without exploring with family first. | *Need for Education*  *Misinterpretation, Misdiagnosis, and Stigma*  *Communication between Healthcare Professionals and Parents* |
| Quantitative Studies | | | | | |
| Alalwan (2022)  Saudi Arabia | Survey | 59 HCP and 316 medical students   - 253 males, 122 females - 333 Saudi, 42 non-Saudi | Moderate | - Measuring knowledge of HCPs and medical students - Majority scored between 60%-80%. - Family physicians displayed greater knowledge than general practice HCPs, paediatricians. - HCPs showed greater knowledge for related conditions more so than tic disorders. - Knowledge did not vary significantly between year of study (Year 1-4). - Most had not heard of HRT but were interested to learn more. | *Need for Education*  *Misinterpretation, Misdiagnosis, and Stigma* |
| Ganos et al. (2021)  72 countries across all continents | Survey | 346 HCPs who are members of the Movement Disorder Society (MDS)   - ≤5 years’ experience = 101 (29%)   6–10 years’ experience = 69 (20%)  11–15 years’ experience = 60 (17%)  16–20 years’ experience = 41 (12%)  ≥21 years’ experience = 74 (22%) | Moderate | - HCPs generally felt confident in their beliefs that tics are habitual, and the premonitory urge was a prerequisite to diagnosis. - Diagnosis was made predominantly by clinical evaluations along, with less than half using standardised scales. - Most HCPs screened for comorbidities. - Most HCPs would request other testing methods (e.g., neuroimaging, lab assessments, urine tests) if unsure. - Behavioural therapy (e.g., CBIT, psychotherapy) as preferred as first line therapy, but acknowledge limited trained therapists. Medications used regularly and differ between adults and young people. - Educating families was most commonly rated as a highly effective intervention. - Financial resources of families, referral sources, additional psychiatric symptoms rated as most common challenges for patients. | *Need for Education*  *Misinterpretation, Misdiagnosis, and Stigma* |
| Katona (2013)  UK | Survey | 94 Medical students   - 65 females, 29 males - 12 in Year 1, 17 in Year 2, 45 in Year 3, 11 in Year 4, 9 in Year 5 | Moderate | - 20% would not want their child marrying someone with TS. - 5% would not want their child to be friends with someone with TS. - The appearance of a celebrity on a game show increased awareness of tics but not known whether this generated positive or negative opinions. - However, fewer students knew someone personally with tics than they did with epilepsy. - Most common answer was ‘unknown’ when asked about the causes of tics. | *Need for Education*  *Misinterpretation, Misdiagnosis, and Stigma* |
| Marcks et al. (2004)  USA | Survey | 67 HCPs   - 32 Psychologists, 30 Family Practitioners, 4 Psychiatrists, 1 Neurologist - 41 males, 26 females - 62 Caucasian, 3 Asian, 2 Hispanic - <5 years - 3%   5-10 years - 26.9%  11-20 years - 35.8%  >20 years - 34.3% | High | - Occupation was not statistically significant in determining knowledge of tics. Slight trend towards increased knowledge of HCPs with working experience of tics but not statistically significant, except for knowledge of rebound effect. - HCPs were mostly correct in their knowledge of tics. - Large majority aware of tic-related stigma. - Poor understanding of diagnostic criteria for tic disorders. - Greater knowledge of national support groups than local groups, with HCPs with more experience having better knowledge of these. - More psychologists than physicians thought psychologists had a role in treatment of tics. Physicians thought psychologists played a role in schools or treating comorbidities. - Few HCPs had heard of HRT, with an even smaller portion of HCPs knowing how to implement. Psychologists more interesting in learning more about HRT than physicians. | *Need for Education*  *Misinterpretation, Misdiagnosis, and Stigma* |
| O’Hare (2016)  Australia | Survey | 86 caregivers of people with tics and a control group of 104 caregivers of people with no developmental disorder diagnosis (N=190)  TS Group   - 71 males, 12 females (3 unaccounted) - 74 Caucasian, 6 other, 3 Asian, 1 unaccounted, 2 Indigenous Australian - 6 low income, 26 Low to middle income, 76 middle and above income   Control Group   - 79 males, 29 females - 107 Caucasian, 1 other - 8 low income, 19 low to middle income, 55 middle and above income | High | - Comorbidities often result in misdiagnosis. - A small majority of respondents said they did not have difficulty accessing support services. - Large majority reported dissatisfaction with the support offered by healthcare services and schools. - Most received treatment from allied health professional for tics.   Just under half of respondents had experienced tic-related stigma. | *Need for Education*  *Misinterpretation, Misdiagnosis, and Stigma* |
| Wand et al. (1992)  Canada | Survey | 462  274 caregivers  123 people with tics  30 other people within household  34 combinations of young people and caregiver   - 423 females, 39 males | Low | - Misdiagnosis was common amongst respondents. - Majority had not received any information about tics, with most people gathering information from non-medical sources such as the media and friends/family. | *Need for Education*  *Misinterpretation, Misdiagnosis, and Stigma* |
| Wellen et al. (2023)  USA | Survey | 186 parents and caregivers  (330 consented, 144 excluded)   - 173 females, 7 males, 6 other   YP with TD (parent reported)   - 121 males, 65 females - 166 Caucasian, 13 multiracial, 2 American Indian or Alaskan Native, 2 Asian, 1 Black, 1 other - 29 <$49,999, 60 $50,000-$99,999, 90 $100,000->$150,000 | High | - Most caregivers reported seeing multiple health providers for tics. - Neurologists and psychiatrists were seen as most knowledgeable about tics. - Those with comorbidities were more likely to have received treatment for tics. - While 84% were seen by a HCP within 3 months, access to local tic specialists were rare and was considered most challenging barrier. - Financial costs made it difficult for caregivers. - 10% reported misdiagnosis for ADHD, allergies, or eye problems. - Some were told their tics would go away and not to worry, or to mindfully watch symptoms. - Primary care HCPs and paediatricians having more knowledge was considered the most important improvement in tic support. | *Need for Education*  *Misinterpretation, Misdiagnosis, and Stigma*  *Communication between Healthcare Professionals and Parents* |
| Mixed Methodology Studies | | | | | |
| Cuenca et al. (2015)  UK | Survey and interviews | Survey with 295 parents of people with tics (358 consented, 65 data excluded) and interviews with 42 young people with tics  Caregiver   - 237 mothers, 18 fathers, 2 other - 106 completed higher education, 85 completed further education, 58 completed secondary school, 7 did not complete compulsory education   YP with tics   - 32 males, 10 females - 36 Caucasian, 6 mixed ethnicity | High | - Lack of understanding/knowledge of TS by HCPs - Almost 15% of parents report having limited or no information provided about TS. - Difficulties accessing specialist care due to delays, cancellations, and insufficient funding. - 32% of parents reported difficulty accessing referral by GP due to limited knowledge. - More knowledgeable professionals helped parents feel better equipped to manage and understand diagnosis. - Positive and negative experiences of medications and behavioural interventions. | *Need for Education*  *Misinterpretation, Misdiagnosis, and Stigma* |
| Marino et al. (2023)  UK | Survey with open and closed questions | 33 adults with tics and 94 parents of young people with tics  YP with tics (parent reported)   - 51 males, 42 females, 1 non-binary - 86 Caucasian, 7 mixed ethnicity, 1 Asian   Adults with tics   - 20 females, 10 males, 3 non-binary - Ethnicity not reported | High | - Few primary care HCPs considered tics at first consultation. - Primary care HCPs demonstrated more diagnosis uncertainty in adults than young peoples. - Most received secondary care referral. - More than half were dissatisfied with support due to minimisation/dismissal, misinterpreted symptoms, difficulty with referrals. - Just over a quarter of respondents saw a tic specialist and was more common in adults than children. - Most 3-6 months secondary care wait times, some longer than a year. Some then received no support and had to go back to GP to be told there was nothing that could be done. - Just over 20% reported primary care HCP being compassionate, collaborative, and active in referral process. | *Need for Education*  *Misinterpretation, Misdiagnosis, and Stigma*  *Communication between Healthcare Professionals and Parents* |
| Rodin et al. (2021)  Uganda | Survey and semi-structured interviews | 152 HCPs and students  21 Medical doctors  52 Nurses  22 Psychiatric clinical officers  30 Psychologist  87 Social workers  2 Occupational therapists  14 Medical students  4 undisclosed  6 participants consented to interview | High | - Most demonstrate a lack of knowledge of tics and thought training was inadequate or non-existent. - Few HCPs had met someone with tics. - Families do not always come forward about tics due to stigma, cultural interpretation of tics (e.g., possession), or not seeking tics as a concern. - Tics misdiagnosed as seizure-related, other neurodevelopmental disorder, anxiety, or as person’s mannerisms/attention seeking. - Less than half thought tic disorder diagnosis was importance. - Just under half were confident in making a tic disorder diagnosis. - Specialists were specific to one area. - All interviewees desired to learn more about tics and offered recommendations for educational tool. | *Need for Education*  *Misinterpretation, Misdiagnosis, and Stigma* |
| Stacy et al. (2023)  USA | Survey with open and closed questions | 57 Paediatric Neurologists   - Mean 25 years' experience   42 Paediatric Psychiatrists   - Mean 25 years' experience   81 General Neurologists   - Mean 27 years' experience   120 General Psychiatrists   - Mean 29 years' experience   67 Caregivers   - 35 females, 32 males   YP with tics (parent reported)  48 males, 19 females | High | - Difficulty in diagnosis due to lack of knowledge, misinterpretation of symptoms, and mixed communications. - Knowledge of best treatment for tics and how/when to implement not consistently understood by HCPs. - HCPs display mixed recognition of stigma associated with tics, and whether to query mood symptoms at consultation. - Parent-rated scores of HCP knowledge generally corresponded with HCP-rated confidence of tic knowledge. | *Need for Education*  *Misinterpretation, Misdiagnosis, and Stigma*  *Communication between Healthcare Professionals and Parents* |

### Appendix C

*A summary of the themes and subthemes identified in the review*

| Themes | Subthemes |
| --- | --- |
| ***Need for Education and Effective Implementation***  Most common theme that suggested HCPs quality and quantity of training was inadequate. | The impact of limited knowledge Knowledge of tics to be ‘moderate’ to ‘good’ but HCPs lack confidence, negatively impacting support. HCPs made recommendations for content of future educational resources. Consequently, families were dissatisfied and saw more HCPs overall. This impacted work life and finances. |
|  | Limited support and unclear referral process More focus placed on comorbidities rather than the tics. Little information of tic management given to patients. Medication primary treatment due to limited knowledge of behavioural therapies, but HCPs were interested to learn more about HRT. GPs (Primary care) demonstrated limited understanding of secondary care pathway. |
| *Misinterpretation, Misdiagnosis and Stigma* Identified in all studies and cultures in this review. | Misinterpretation and misdiagnosisIn some cases, HCPs perception of TD being rare may result in symptom misinterpretation or misdiagnosis. Motor tics most recognised by HCPs. Overestimation of coprolalia in tic cases. Varied understanding of tic remission and prevalence of premonitory urge. |
|  | Stigma and misconceptions Tic symptoms were trivialised by both HCPs and families. Depending on cultural belief system, stigma would either promote or impede help-seeking behaviour. |
| *Communication between Healthcare Professionals and Families* Featured in four of thirteen studies. Families had a stronger preference for joint decision-making than HCPs, and complex language obstructed the clinical relationship developing. Even if HCPs were perceived to have limited knowledge, families appreciated engaged and compassionate HCPs who would follow up on referrals. |  |

### Appendix D

*A graph showing the distribution of included studies by publication date and participant sample size.*
